# Genomic Organization, Transcriptomic Analysis, and Functional Characterization of Avian α- and β-Keratins in Diverse Feather Forms

**DOI:** 10.1093/gbe/evu181

**Published:** 2014-08-24

**Authors:** Chen Siang Ng, Ping Wu, Wen-Lang Fan, Jie Yan, Chih-Kuan Chen, Yu-Ting Lai, Siao-Man Wu, Chi-Tang Mao, Jun-Jie Chen, Mei-Yeh Jade Lu, Meng-Ru Ho, Randall B. Widelitz, Chih-Feng Chen, Cheng-Ming Chuong, Wen-Hsiung Li

**Affiliations:** ^1^Biodiversity Research Center, Academia Sinica, Taipei, Taiwan; ^2^Department of Pathology, Keck School of Medicine, University of Southern California; ^3^Jiangsu Key Laboratory for Biodiversity and Biotechnology, College of Life Sciences, Nanjing Normal University, China; ^4^Institute of Ecology and Evolutionary Biology, National Taiwan University, Taipei, Taiwan; ^5^Molecular Biology of Agricultural Sciences, Taiwan International Graduate Program, Academia Sinica, Taipei, Taiwan; ^6^Graduate Institute of Biotechnology, National Chung Hsing University, Taichung, Taiwan; ^7^Department of Animal Science, National Chung Hsing University, Taichung, Taiwan; ^8^Center for the Integrative and Evolutionary Galliformes Genomics (iEGG Center), National Chung Hsing University, Taichung, Taiwan; ^9^Department of Ecology and Evolution, University of Chicago

**Keywords:** keratin, feather, skin appendage, evolution, transcriptome, RNA-seq, chicken, zebra finch, in situ hybridization

## Abstract

Feathers are hallmark avian integument appendages, although they were also present on theropods. They are composed of flexible corneous materials made of α- and β-keratins, but their genomic organization and their functional roles in feathers have not been well studied. First, we made an exhaustive search of α- and β-keratin genes in the new chicken genome assembly (Galgal4). Then, using transcriptomic analysis, we studied α- and β-keratin gene expression patterns in five types of feather epidermis. The expression patterns of β-keratin genes were different in different feather types, whereas those of α-keratin genes were less variable. In addition, we obtained extensive α- and β-keratin mRNA in situ hybridization data, showing that α-keratins and β-keratins are preferentially expressed in different parts of the feather components. Together, our data suggest that feather morphological and structural diversity can largely be attributed to differential combinations of α- and β-keratin genes in different intrafeather regions and/or feather types from different body parts. The expression profiles provide new insights into the evolutionary origin and diversification of feathers. Finally, functional analysis using mutant chicken keratin forms based on those found in the human α-keratin mutation database led to abnormal phenotypes. This demonstrates that the chicken can be a convenient model for studying the molecular biology of human keratin-based diseases.

## Introduction

For birds, feathers play a crucial role in heat retention, mate attraction, protection, flight, etc. Feathers can have such diverse functions because they form different structures to adapt to functional needs in different body parts or at different times of their life (Chuong et al. 2012). There are specific feather types in different body regions, and there are different branching morphologies in different parts of the same feather (Lin et al. 2013). The feather is a unique morphological innovation which might have originated from modifications of reptilian scales (Greenwold and Sawyer 2010) and evolved in nonavian dinosaurs and basal birds (Prum and Brush 2002; Wu et al. 2004; Xu et al. 2010). The successful diversification of feather forms presumably has contributed significantly to the rapid and extensive radiation of birds to become the dominant terrestrial vertebrate.

The major components of feathers are α- and β-keratins, which are encoded by multigene families (Alibardi and Toni 2008). The emergence of novel, lineage-specific morphological features can be attributed to expansion of these gene families (Conant and Wolfe 2008). This has been proposed as a critical evolutionary mechanism that drives molecular diversity (Ohno 1970). For instance, the independent origin of hair and nails in mammals and baleen in whales might have been led by the expansion of α-keratin genes (Vandebergh and Bossuyt 2012). Large-scale expansions of β-keratin genes in birds and turtles were proposed to be associated with the innovation of the feather and turtle shell (Greenwold and Sawyer 2010; Li et al. 2013).

In birds, five β-keratin gene subfamilies (claw, feather, feather-like, keratinocyte, and scale) have been classified by sequence heterogeneity and tissue-specific expression (Presland et al. 1989; Presland, Whitbread, et al. 1989; Whitbread et al. 1991; Greenwold and Sawyer 2010). Previous genome-wide comparative analyses in zebra finch and chicken identified several clusters of β-keratin genes; the largest two are on chromosomes 25 (Chr25) and 27 (Chr27) (Greenwold and Sawyer 2010). The acquisition of new β-keratin genes in birds was most likely correlated with functional diversification of these genes. New β-keratin genes in the expanded β-keratin multigene family might have been selected for novel functions in evolved skin appendages such as the feather of birds and the plastron and carapace of turtles. However, mapping the keratin genes within the avian genome has been extremely challenging due to the high similarity between duplicated genes.

Although the expansion and radiation of the avian β-keratin genes could have contributed to the evolution of feathers and the diversification of birds, little work has been carried out to characterize their expression profiles in different feather parts and types. Coordinated expression of the acidic and basic keratins, which are encoded by the Type I and Type II α-keratin gene clusters, is also essential for skin appendage development. Characterization of the genomic organization is helpful for understanding the evolution and regulation of α- and β-keratin genes. Knowledge of the timing and tissue expression of copious α- and β-keratin genes would allow us to associate feather shape with the specific keratins produced to form the ramus, barbules, rachis, and calamus in various feather types.

The availability of transcriptomic analysis tools and avian whole-genome sequences provides an excellent opportunity to study evolutionary processes and gene expression patterns that potentially account for morphological variations. In this study, we aim to identify α- and β-keratin genes involved in the formation of different types of feathers at different developmental stages. We search for and annotate the α- and β-keratin sequences in the new chicken genome assembly, and analyze the expression profiles of the α- and β-keratins during the development of different feather types by RNA-seq and by in situ hybridization. Finally, we conduct functional analysis using mutant chicken α-keratin forms based on those found in the human α-keratin mutation database.

## Materials and Methods

### Ethics Statement

All the animals used in this study were processed following the approved protocol of the Institutional Animal Care and Use Committees of National Chung Hsing University (Taichung, Taiwan) and University of Southern California (Los Angeles, CA).

### Eggs and Animals

For the functional study, pathogen free fertilized eggs were purchased from SPAFAS (Preston, CT). Some of these eggs hatched and the chickens were used for functional studies on adult feather follicles. For total RNA extraction, we used Taiwan County Chicken (TCC_L2) breed chicken for wing flight feather and white leghorn for body contour feather. For section in situ hybridization, we used white leghorn chickens to avoid blocking the signal from in situ hybridization by the pigmentation. The contour feather and flight feather shapes between these two chicken breeds are similar.

### Paraffin Section and Staining

Control or gene misexpressed feathers were fixed in 4% paraformaldehyde at 4 °C overnight for immunohistochemistry and 7-μm paraffin sections were prepared followed by procedures described by Jiang et al. (1998). PCNA and AMV-3C2 antibodies are from Chemicon (CBL407) and the Hybridoma Bank, respectively.

### mRNA *in situ* Hybridization

To generated specific α- and β-keratin antisense RNA probes, we used the 3’-UTR (untranslated region) of mRNA as polymerase chain reaction (PCR) target. PCR primers are listed in supplementary table S4, Supplementary Material online. We also generated a common Type I α-keratin probe, a common Type II α-keratin probe, and a common β-keratin probe, using the conserved coding region as PCR target (supplementary table S4, Supplementary Material online). The PCR product was inserted into the p-drive plasmid (Qiagen). Antisense probe was made to detect the mRNA expression by section in situ hybridization. Nonradioactive in situ hybridization was performed according to procedures described in Chuong et al (1996).

### Construction of RCAS-KRT5 Mutant Forms

We used PCR to clone chicken KRT5. We applied the QuikChange Lightening Site-Directed Mutagenesis Kit (Agilent Technologies, Santa Clara, CA) to generate KRT5-N183Δ (Kang et al. 2010) and KRT5-R464_A468Δ (Kemp et al. 2005) as well as the QuikChange Lightening Multi Site-Directed Mutagenesis Kit (Agilent Technologies) to generate KRT5-V170_K191Δ (Rugg et al. 1999) (supplementary fig. S7 and table S5, Supplementary Material online). The DNA fragments were cloned into the pCR8/GW/TOPO Gateway entry vector (Invitrogen, Carlsbad, CA) and sequenced. An LR recombination reaction was performed to transfer the cDNAs to a Gateway compatible RCASBP-Y DV vector (Loftus et al. 2001). Virus was made according to Jiang et al. (1998) and concentrated by ultracentrifugation.

### Functional Studies of *KRT5*

For adult feathers, about 100 μl of virus was injected into the empty follicles after plucking the primary flight feathers in the left wing. The feathers on the right wing were collected at the same time as the controls. Feather follicles from a different chicken injected with RCAS-GFP were used as an alternative control. Feather morphogenesis was observed after 1–2 months of regeneration.

### Feather Regeneration and Collection

We collected regenerating pennaceous and plumulaceous portions of body contour feathers, distal and proximal portions of primary flight feathers, and the calamus of primary flight feathers. Around 50 contour feathers from the middle back of the body were plucked and then collected after 14 (early growth phase), 42 (late growth phase) and primary flight feathers were plucked and then collected after 144 (early growth phase), 42 (middle growth phase), or 56 days (late growth phase). At the collection points, regenerated feathers were directly plucked and the whole single feather follicle tissue was isolated and preserved in RNALater solution (Ambion) immediately. To confirm the type of feather, we also fixed some whole single follicles in 4% paraformaldehyde at 4 °C for sectioning (supplementary fig. S2, Supplementary Material online). The body contour feathers and wing flight feathers from white leghorn chicken are collected using the same time frame for section in situ hybridization purposes.

### Total RNA Isolation

The feather follicle tissue was incubated at 4 °C overnight for penetration by RNALater solution and then transferred to −20 °C before further isolation of total RNA. Epithelium was dissected from the follicle tissue and separated from the mesenchyme in Calcium-Magnesium Free Saline (CMFS 2X) on ice (Chuong 2000). Total RNA from feather epithelium was insolated using the RNEasy Plus Mini Kit (Qiagen, Hilden, Germany) with an additional on-column DNase treatment recommended by the manufacture (Qiagen). The 15-min DNase treatment was carried out at room temperature by mixing 10 μl DNase and 70 μl RDD buffer and applied to the RNA-binding column after the first wash. The RNA quantities and qualities of each individual were analyzed by NanoDrop (Thermo Scientific, Waltham, MA) and BioAnalyzer II (Agilent Technologies). If all samples from the same litter passed the quality control (RNA integrity number > 8.0), 10 µg of total RNA from each sample would be pooled to reach a final of 30 µg total RNA for sequencing for each sample.

### RNA Sequencing

For paired-end mRNA-seq library preparation, we used Illumina TruSeq mRNA-seq kits. A total of 5 μg total RNA was used as input for mRNA enrichment by oligo-dT beads followed by cation-catalyzed fragmentation for 7 min at 94 °C. The mRNA fragments were then converted into double-stranded cDNA by random priming followed by end repair and A-tailing. The fragmented cDNAs were then ligated to the paired-end adaptors, followed by ten cycles of PCR amplification. The libraries were purified by Ampure beads (Beckman Agencourt, Brea, CA) to remove small fragments. The absolute concentrations of the libraries were determined by Qubit fluorometry (Invitrogen) and BioAnalyzer High Sensitivity DNA Kit (Agilent Technologies). Each mRNA-seq library was loaded in one lane of flow cell and paired-end 2 × 101 nt sequencing was conducted on Illumina HiSeq2000, totaling five lanes of data for the five tissue types (average one lane per tissue type). Library preparation and Illumina sequencing was conducted by High Throughput Sequencing Core Facility, Biodiversity Research Center, Academia Sinica, Taiwan.

### Analysis of Paired-End Reads

Low-quality bases and reads were removed by three criteria: 1) The consecutive bases from the end of a read with a default low-quality score of 2 (Phred score of 2 or Q2 [2]), 2) the bases from the beginning of a read until all of the scores of the first 20 remaining bases were at least Q20 (the base call error rate of ∼1%), and 3) the trimmed reads with less than 60 remaining bases. (Phred score is a general metric for the accuracy of a sequencing platform.) The Q2 indicator does not give a specific error rate, but rather indicates a specific portion of the read that should not be used in further analyses. We trimmed all the paired-end sequencing reads from both ends of each cDNA fragment to 90 bp to reduce sequencing errors. The processed reads were mapped to the chicken genome and the working gene set, using Tophat version 1.3.3 (http://ccb.jhu.edu/software/tophat/index.shtml, last accessed September 3, 2014) (Trapnell et al. 2009), and its embedded aligner Bowtie version 0.12.7 (http://bowtie-bio.sourceforge.net/index.shtml, last accessed September 3, 2014) (Langmead et al. 2009). Each read was aligned by the “-n” policy, and at most ten hits were allowed. The normalized expression levels of genes, measured in fragments per kilobase of exon per million fragments mapped (FPKMs) (Mortazavi et al. 2008), were calculated using Cufflinks version 2.0.2 (http://cufflinks.cbcb.umd.edu/, last accessed September 3, 2014) (Trapnell et al. 2013). Only those pair-end reads mapped to the genome without mismatch were used for subsequent analyses. We first categorized mappable fragments into two groups: “Unique” fragments, each of which was mapped to a single position in the genome, and “multiple-hit” fragments, each of which was mapped to more than one position in the genome. To calculate the expression levels, unique fragments were assigned to an individual gene first for initial abundance estimation, and the multiple-hit fragments were then redistributed to those genes based on the relative abundances of uniquely mapped fragments. Total mappable fragments on each chromosome were calculated by SAMtools (Trapnell et al. 2010; Roberts et al. 2011).

### Multivariate Analyses

Prior to statistical analyses performed with R v2.15.3 (R Development Core Team 2011), raw read counts were normalized by FPKM and log_2_ transformed. A heat map was generated using the heatmap.2 function in the “gplots” package; principal component analysis (PCA) was performed on the covariance matrix f using a custom R script based on the “prcomp” R package.

### Identification of Differentially Expressed Genes

We used the nonparametric method to identify differentially expressed genes (DEGs) between two samples (Tarazona et al. 2011). Here, we set the *q* value (differentially expression probability) in the method to be 0.75 and require at least a 2-fold change in RPKM between the two samples (at least 4-fold for β-keratin genes). In each comparison, we conducted the Hypergeometric Test to calculate the *P* value of the enrichment of DEGs in a particular gene set (α- or β-keratin genes) compared with the background (all gene sets, which include 17,214 genes) and also the Fisher Exact Test to test whether the odds ratio of the DEGs between two gene sets (α- and β-keratin genes) significantly deviates from 1.

### Identification of α- and β-Keratin Genes

The Type I/II α-keratin and β-keratin nucleotide sequences, amino acid sequences, and unique features associated with the keratin genes were obtained from Ensembl and National Center for Biotechnology Information (NCBI). The BLAT searches (Kent 2002; Bhagwat et al. 2012) implemented in the University of California Santa Cruz Genome Browser database (http://genome.ucsc.edu, last accessed September 3, 2014) as used to search the ICGSC Gallus_gallus-4.0 (GCA_000002315.2) genome sequence for additional β-keratin genes. Proteins sequences or predicted proteins were extracted and used in subsequent BLAT searches that were reiterated until no new keratins were found. We included all β-keratin genes that had both reasonable start and stop codons predicted using NCBI ORF Finder (http://www.ncbi.nlm.nih.gov/gorf/gorf.html, last accessed September 3, 2014). Translated protein sequences from additional β-keratin genes were confirmed to have the avian keratin domain using InterProScan (Zdobnov and Apweiler 2001; Quevillon et al. 2005). In addition to the cluster of β-keratins identified by Greenwold and Sawyer (2010, 2013), our analysis revealed additional genomic loci containing feather β-keratins. For α-keratin genes, we used AUGUSTUS (Stanke and Morgenstern 2005; Stanke et al. 2006) or GenScan (Burge and Karlin 1998) to predict the coding sequences (CDS). If more than one CDS of α-keratin genes were predicted, we aligned the identified CDS and checked the predicted protein sequences to identify the most conserved pattern.

### Phylogenetic Analysis

Alignments were done using the program CLUSTALW Multiple Sequence Alignment Program (Thompson et al. 1994) with default parameters. Visual inspection confirmed an adequate alignment. Tree reconstruction was done using a total of 152 taxa, which included all current putative β-keratins. As an outgroup for tree reconstruction, three β-keratin nucleotide CDS were used from *Crocodylus niloticus* (Nile crocodile) and were obtained from NCBI with the GenBank numbers: 215541571, 215541573, and 187942180 (Dalla Valle et al. 2009). The Type I and Type II α-keratin genes using Amphioxus (*Branchiostoma floridae* and *Branchiostoma lanceolatum*), and Ciona (*Ciona intestinalis*) intermediate filament genes were used as an outgroup for α-keratin gene tree. We used MEGA6 (Tamura et al. 2013) to select the best DNA model using a maximum-likelihood (ML) method. The phylogenetic trees were inferred using the ML method based on the Tamura–Nei model (Tamura and Nei 1993). The percentage of trees in which the associated taxa clustered together is shown next to the branches. Initial tree(s) for the heuristic search were obtained by applying the neighbor joining method to a matrix of pairwise distances estimated using the Maximum Composite Likelihood approach. A discrete Gamma distribution was used to model evolutionary rate differences among sites. The tree is drawn to scale, with branch lengths measured in the number of substitutions per site. All positions with less than 95% site coverage were eliminated. That is, fewer than 5% alignment gaps, missing data, and ambiguous bases were allowed at any position. Evolutionary analyses were conducted in MEGA6 (Tamura et al. 2013).

## Results

### Structural Analysis of Feathers at Different Developmental Stages

**Fig. 1.—:**
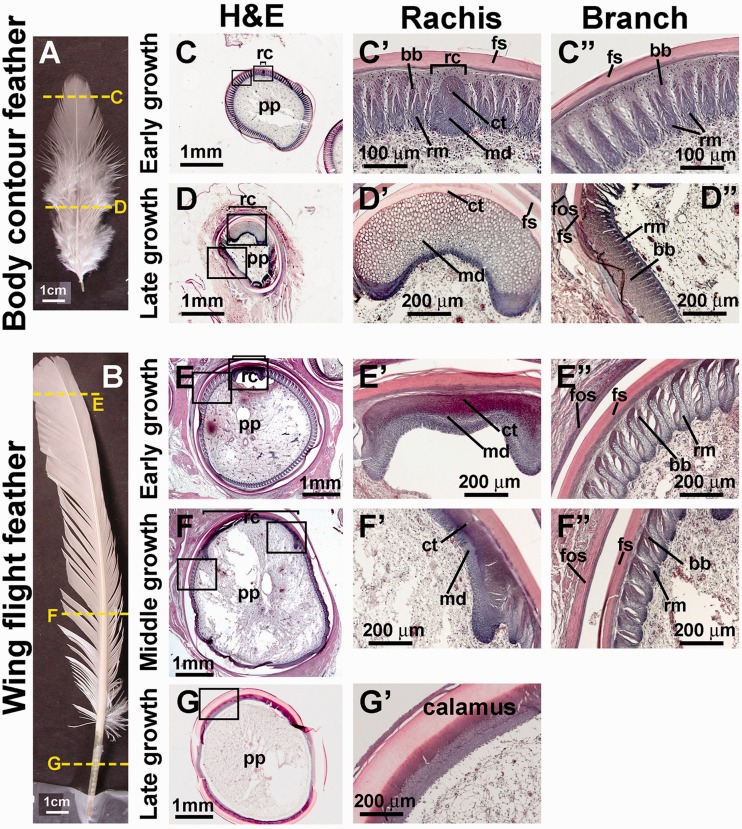
Structures of body contour feathers and wing flight feathers at different growth phases. Mature contour feather (*A*) and flight feather (*B*). There is a temporal order along the proximal–distal feather axis with the distal portion formed earlier (Lin et al. 2013). (*C, D*) H&E staining of cross sections of contour feathers at early and late growth phases. (*E–G*) H&E staining of cross sections of flight feathers at early, middle, and late growth phases. (*C’—F’*) Rachis region with higher magnification. (*C”–F”*) Barb branch region with higher magnification. (*G’*) Calamus with higher magnification. The dotted line in panels (*A*) and (*B*) indicates the position for sectioning. bb, barbule; ct, cortex; fs, feather sheath; fos, follicle sheath; md, medulla; pp, pulp; rc, rachis; rm, ramus.

Feathers at different parts of the body display different shapes, textures and stiffness, which are related to their functions for protection, thermoregulation, or flight. We focus on body contour and wing flight feathers (fig. 1*A* and *B* and supplementary fig. S1, Supplementary Material online). Body contour feathers have a pennaceous vane on the distal upper portion and a plumulaceous, fluffy part in the proximal lower portion. The pennaceous vane functions display and makes the body aerodynamically stream-lined for flight, whereas the plumulaceous part is used to provide warmth. In body contour and wing flight feathers, the rachis is thinner at the distal end and thicker at the proximal end to support the vanes. The calamus in body contour feathers is short. Compared with contour feathers, flight feathers have a larger pennaceous vane and a longer and thicker rachis. Wing flight feathers also have a longer calamus for insertion deeper into the follicle and anchor more securely to sustain its aerodynamic function.

We collected regenerating feather follicles at different regeneration time points, so they would be in the formative time of different feather structures. For body contour feathers, two time points (day 14 for the early growth phase and day 42 for the late growth phase) were used (fig. 1*A*). For wing flight feathers, three time points (day 14 for the early growth phase, day 42 for the middle growth phase, and day 56 for the late growth phase) were used (fig. 1*B*). We compared H&E (Hematoxylin and Eosin) staining from the paraffin sections of different feathers at different time points (fig. 1*C–G’*). Early growth phase contour feathers show a tiny rachis and numerous pennaceous barb ridges (fig. 1*C* and *C’*). Only a few barb ridges show the barbule (fig. 1*C”*). Body contour feathers in late growth phase show a much wider rachis with dominant medulla (fig. 3*D* and *D’*). The barb ridge includes a tiny ramus and numerous plumulaceous barbules (fig. 1*D”*). Early and middle growth phase flight feathers show different rachis sizes (fig. 1*E–F’*) and smaller pennaceous barb ridges in the middle growth phase (fig. 1*F”*, compared with 1*E”*). A ring-shaped calamus without barb ridges appears in the late growth phase (fig. 1*G* and *G’*).

### Genome Search and Evolutionary Analysis of α-Keratin Genes

We applied BLAT to screen for α-keratin genes in the current chicken genome assembly, the ICGSC Gallus_gallus-4.0 (GCA_000002315.2) (November 2011). We also considered the annotation of some genes in GenBank and applied gene prediction software. We found 33 putative α-keratin genes, four more genes than reported in a recent study (Vandebergh and Bossuyt 2012) but the same number as found in an earlier study based on an older genome assembly (Galgal3) (Zimek and Weber 2005). The two large gene families (Type I and Type II) are made up of 54 genes in human and mouse genomes (Hesse et al. 2004). Chicken α-keratin genes have been poorly studied in the past and no one has tried to relate all individual α-keratin genes between chicken and mammals yet. We tried to compare the chicken α-keratin genes with human α-keratin genes, renaming the chicken gene to match its putative human homolog if necessary.

**Fig. 2.—:**
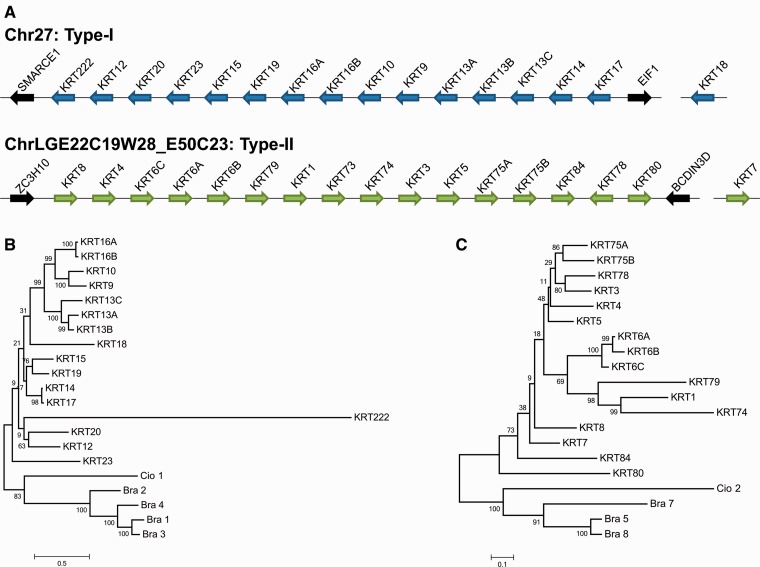
Type I and Type II α-keratin genes of *Gallus gallus*. (*A*) The genomic organization of the Type I and Type II α-keratin genes of *G. gallus*. Arrows indicate the transcriptional orientation of the coding regions. (*B*) Paralogous evolutionary analysis of chicken Type I α-keratin genes. (*C*) Paralogous evolutionary analysis of chicken Type II α-keratin genes. The trees were constructed using the ML method. The bootstrap values are listed for each major branch. Chordate α-keratin genes are presented as the outgroup.

For both Type I and Type II α-keratin clusters, our analyses showed that there are 15 genes in cluster one, which is on Chr27, and 17 genes in cluster two, which is on ChrLGE22C19W28_E50C23. One of the Type II α-keratin genes is situated on an unassembled contig which is probably linked to ChrLGE22C19W28_E50C23. One Type I α-keratin gene, homologous to human *KRT18*, is located on an unassembled contig and likely not linked to the Type I cluster. In humans and mice, *KRT18* is not contained in the Type I cluster and instead locate beside the Type II cluster (Hesse et al. 2004). The chicken Type I α-keratin cluster is approximately 129 kb in size and has a density of one gene per 8.6 kb in average, whereas the Type II α-keratin cluster is approximately 202 kb in size and has a density of one gene per 11.9 kb in average. The Type I cluster was situated between the flanking genes *SMARCE1* and *EIF1*, whereas the Type II cluster was situated between *BCDIN3D* and *ZC3H10* in the chicken genome (fig. 2*A*). The left flanking gene of Type II α-keratin cluster is instead *EIF4B* in the zebra finch genome (supplementary fig. S3, Supplementary Material online). Our search result for chicken and zebra finch α-keratin genes is summarized in supplementary tables S1 and S2, Supplementary Material online, respectively, which also provide the chromosomal locations of the genes and their orientation as well as sequences.

We reconstructed the phylogenetic relationship among these Type I and Type II α-keratin genes using Amphioxus (*B. floridae* and *B. lanceolatum*), and Ciona (*C. intestinalis*) intermediate filament genes as the outgroups. We found that several α-keratins may be recent duplicates (*KRT16A/KRT16B* and *KRT75A/KRT75B*). *KRT16A/KRT16B* and *KRT75A/KRT75B* can also be found in the zebra finch genome but only single ortholog copies of these genes could be found in the American alligator genome (allMis0.2/allMis1), suggesting that they were duplicated in a common avian ancestor (supplementary fig. S4, Supplementary Material online).

### Genome Search and Evolutionary Analysis of β-Keratin Genes

As β-keratin genes are clustered as tandem arrays at several chromosomal locations, nearly complete and nonredundant β-keratin gene inventories can be achieved from an in-depth screening and examination of the chicken genome. Chicken β-keratins can be subdivided into multiple phylogenetic clades, which are associated with different genomic locations. Greenwold and Sawyer (2010, 2013) found 133 β-keratin genes in the WUGSC2.1/galGal3 genome sequence, whereas we identified 149 β-keratin genes in the new chicken genome assembly. These 149 β-keratin genes with their sequences, chromosomal locations, and orientations are summarized in supplementary table S3, Supplementary Material online.

Among the newly found β-keratin genes, several are similar to the β-keratins from cultured keratinocytes (Presland et al. 1989; Presland, Whitbread, et al. 1989). Previously, only 11 keratinocyte-β-keratins had been detected in the older chicken genome assembly (Greenwold and Sawyer 2013), whereas we now identified 16 unique keratinocyte-β-keratin sequences. Also, we identified eight additional chicken scale-β-keratins to make a total of 18 scale-β-keratins. Moreover, we identified four new claw β-keratins to make a total of 12 chicken claw-β-keratins.

**Fig. 3.—:**
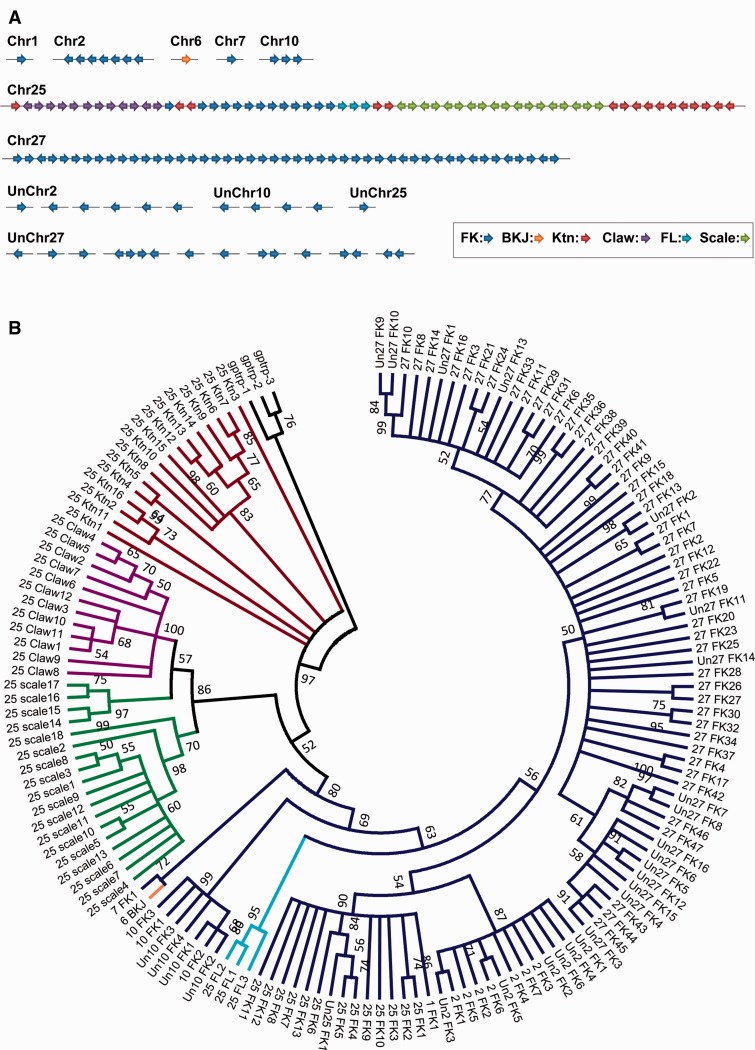
β-keratin genes of *Gallus gallus*. (*A*) The genomic organization of the β-keratin subfamilies of *G. gallus*. Arrows indicate the transcriptional orientation of the coding regions. The six β-keratin subfamilies: β-keratin in *jun*-transformed cells (BKJ), feather-like (FL), feather (FK), keratinocyte (Ktn), scale, and claw are color labeled. Genes on unassembled contigs are also shown. (*B*) Consensus phylogenetic tree of the β-keratin genes of chicken. Three Nile crocodile β-keratin genes are presented as the outgroup with all 149 β-keratin genes found in the chicken genome. The bootstrap values are listed for each major branch when they are above 50%. The subfamilies are colored with the following scheme: Feather-β-keratin: dark blue, feather-like-β-keratin: light blue, BKJ-β-keratin: orange, scale-β-keratin: green, claw-β-keratin: purple, and keratinocyte-β-keratin: brown.

A large number of feather-β-keratin genes in the chicken genome are located on Chr27 (63 genes) and Chr25 (13 genes), whereas some feather-β-keratin genes are present on Chr1 (1 gene), Chr2 (13 genes), Chr7 (1 gene), and Chr10 (6 genes). The gene order of β-keratin genes on Chr25 is basically claw-, feather-, feather-like-, scale-, and keratinocyte-β-keratin in a 5’–3’ direction spanning approximately 256 kb. There are 11 keratinocyte-β-keratin genes located downstream of the scale-β-keratin genes, with five keratinocyte-β-keratin genes scattered in the cluster on Chr25 (fig. 3*A*).

There are seven feather-β-keratin genes on Chr2 (a macrochromosome) spanning approximately 22.6 kb. Six additional feather-β-keratin genes on an unknown chromosome are highly similar with the feather-β-keratin genes found on Chr2 and have not yet been placed in the current build of the chicken genome. We only found one BKJ-β-keratin (β-keratin in *jun*-transformed cells) gene on Chr6, which is similar to the β-keratin isolated from *jun*-transformed quail (*Coturnix japonica*) fibroblast cells (Hartl and Bister 1995), although three were found in an older chicken genome assembly (Galga3). Two similar but incomplete BKJ-β-keratin CDS were found in unassembled contigs. There is a tandem array of 47 feather β-keratin CDS located on Chr27 spanning approximately 577.6 kb. Sixteen additional feather-β-keratin genes on an unknown chromosome are highly similar with the feather-β-keratin genes found on Chr27 and have not yet been placed in the current build of the chicken genome. Having many genes on unassembled contigs may reflect the difficulty of assembling Chr2, Chr10, and Chr27, probably due to highly conserved sequences of keratin genes or to their high GC content.

Chicken β-keratins can be subdivided into multiple phylogenetic clades (fig. 3*B*). The three feather like-β-keratin genes form a monophyletic group and are basal to feather-β-keratins on Chr25, Chr27, and Chr2 in the ML tree (supplementary fig. S5, Supplementary Material online). The ML tree also shows that the sequence on Chr1 sorts with the feather-β-keratins of Chr25. Chr6_BKJ is a sister of Chr10_FK genes. The β-keratin genes on Chr2 form a monophyletic group except that Chr2_FK7 sorts with Chr7_FK1 in the ML tree (fig. 3*B*). The β-keratin genes on Chr2, Chr10, Chr25, and Chr27 form four monophyletic groups, respectively. We also reconstructed a neighbor joining tree and its topology is similar to the ML tree, except that the Chr25_FL genes are basal to all feather β-keratins (supplementary fig. S6, Supplementary Material online).

The claw- and scale-β-keratin genes form sister groups in the ML tree. The keratinocyte-β-keratin genes are basal to all other β-keratin genes, consistent with previous findings (Greenwold and Sawyer 2010, 2011, 2013). The branch lengths of keratinocyte-β-keratin genes are generally longer than other β-keratin genes, suggesting that they might have diverged before the origins of other β-keratin genes, or evolved at a faster rate. Our phylogenetic trees suggest that precursors of keratinocyte-β-keratin genes radiated extensively and at least some of them acquired their function in feather development before the appearance of feather-β-keratin genes. Our gene expression analysis indicates that some of them are expressed in different regions at various developmental stages of feathers. These ancestral β-keratin genes could be the primers to allow further differential radiations of feather types.

### Differential Expression of Keratin Genes in Different Feather Parts

In order to investigate the expression patterns of avian α- and β-keratin genes, we extracted total RNA from five regenerating chicken feather epithelium tissues, including the rachis, feather branches, and feather sheath, to perform RNA-seq analyses. Two of these five samples were from body contour feathers and three were from flight feathers (fig. 1*A* and *B*). One of our goals was to determine which chicken α- and β-keratin genes are expressed in feather epithelium, thereby lending support to their functional annotation.

cDNA libraries with insert lengths ranging from 300 or 400 bp were constructed for each sample. Reads were mapped onto the new chicken genome assembly ICGSC Gallus_gallus-4.0 (GCA_000002315.2). Expression values were calculated for each sample based on the number of FPKM (Mortazavi et al. 2008). If the fragments were multiply mapped on different genes, the multiple-hit fragments were redistributed to those genes based on the initial abundance estimation of uniquely mapped fragments.

**Fig. 4.—:**
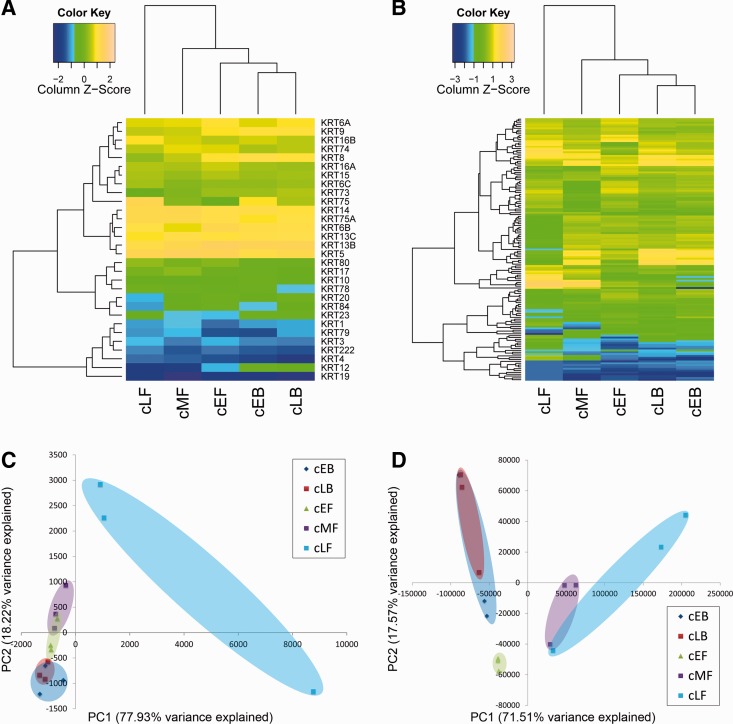
Expression patterns of α- and β-keratin genes in chicken feathers. Heatmap showing relative expression levels of (*A*) α- and (*B*) β-keratin genes among different feather types at different developmental stages. Gene expression data were log_2_ transformed prior to generating the heatmap for direct comparison of data. Colors indicate relative expression (yellow, high expression; black, intermediate; blue, low). PCA of expression patterns of (*C*) α-keratin genes and (*D*) β-keratin genes. Each symbol represents a single sample (*n* = 3 replicate samples per tissue type). Tissue types are indicated by color. cEB, early growth body contour feather; cLB, late growth body contour feather; cEF, early growth flight feather; cMF, middle growth flight feather; LF, late growth flight feather.

We set the threshold value at 0.1 FPKM to define expressed genes. We found 30 identified α-keratin genes and 143 β-keratins to be expressed with an FPKM >0.1 in the chicken feather epithelium in at least one of the samples. Thus, we confirmed that the vast majority of predicted chicken β-keratin genes (95.9%) (fig. 4*B*) and α-keratin genes (90.0%) (fig. 4*A*) is indeed expressed in the feather epithelium. In contrast, the annotations of six predicted chicken β-keratin genes are not supported, as their expression was not detected in our samples. However, as our samples are from feather epithelia, we cannot exclude the possibility that these β-keratin genes are expressed in other types of tissues.

When the gene expression levels among the sampled feather follicles were compared, the β-keratin genes located on Chr2 and Chr6 were expressed at the highest levels in flight feather follicles, which were higher than in contour feather follicles (fig. 4*B*), suggesting that Chr2_FKs may be more important in forming stiff feather structures. In contrast, the expression levels of Chr25_FKs were generally higher in contour feathers than in flight feathers, suggesting that they may be required for softer textures. We also observed that scale- and claw-β-keratin genes were expressed at significant levels in all feather samples. In general, the relatedness of α-keratins in the phylogenetic tree does not show clustering of α-keratins exhibiting expression in feather follicles (fig. 3*A*), suggesting that the function of closely related α-keratin genes was already diversified in the early evolution of feathers (fig. 2*B* and *C*).

We conducted statistical tests and found that significantly higher proportions of DEGs in the α- and β-keratin gene sets compared to the all gene set (table 1). The FPKM estimates for the α- and β-keratin genes in the 15 libraries (five samples, three biological replicates per sample) were subjected to PCA to visualize their expression patterns within and between groups. PCA of the α-keratin RNA-seq data demonstrated that transcriptome profiles of the early growth body contour feathers (EB), late growth body contour feathers (LB), early growth flight feathers (EF), and middle growth flight feathers (MF) were similar to each other with EB and LB overlapping almost completely and EF and MF overlapping partially (fig. 4*C*). LF (late flight feather, calamus only) was well separated from the other groups. Figure 4*C* shows that the first two PCs explain 96.15% of the total variance in the data set with PC1 contributing 77.93% and PC2 18.22%. These observations suggest that the expression patterns of α-keratin genes in the same type of feather are conserved and may not contribute significantly to structural and morphological variations at different developmental stages.

**Table 1:** Percentage of DEGs in All and Particular Gene Sets

Comparison	Tissue Type	Proportion of DEGs in All Gene Sets (%)	Proportion of DEGs in the α-Keratin Gene Set (%)	Percentage of DEGs in the β-Keratin Gene Set (%)
A	EB versus LB	1,053/17,214 (6.12)	6/31 (19.35)*	27/148 (18.24)**
B	EB versus EF	1,393/17,214 (8.09)	12/31 (38.71)**	46/148 (31.08)**
C	EF versus MF	1,164/17,214 (6.76)	10/31 (32.25)**	83/148 (56.08)**^,^†
D	EF versus LF	1,332/17,214 (7.74)	14/31 (45.16)**	101/148 (68.24)**^,†^
E	MF versus LF	702/17,214 (4.08)	16/31 (51.60)**	84/148 (56.76)**

Note.—EB, Early-grow body feather (pennaceous); LB, late-grow body feather (plumulaceous); EF, early-grow flight feather; MF, middle-grow flight feather; LF, late-grow flight feather.

*P < 0.05; **P < 0.001 compared with background (all gene sets); ^†^*P* < 0.05 compared with the α-keratin gene set.

PCA of the β-keratin RNA-seq data demonstrated that transcriptome profiles of the same feather type at different developmental stages were similar to each other with EB and LB overlapping almost completely and MF and LF overlapping partially (fig. 4*D*). EF was well separated from the other groups. In general, samples from different types of feathers were well separated. Figure 4*D* shows that the first two PCs explain 89.08% of the total variance in the data set with PC1 contributing 71.51% and PC2 17.57%. The distal part of flight feather (EF) is softer than the remaining portion of flight feathers, so that the samples of EF were not separated from the body contour feather samples on the PC1 axis. We also found significantly higher proportions of DEGs in the β-keratin gene set than in the α-keratin gene set in two comparisons, when we applied a stricter criterion for identifying DEGs in the β-keratin gene set (table 1). These observations suggest that the expression patterns of β-keratin genes are different in different feather types and may in part explain the structural and morphological variations among feather types. The most obvious differences between the body contour and wing flight feathers are the texture and stiffness, which can be due to different biochemical and biophysical properties of different β-keratins.

The expression patterns of α-keratin genes were similar among different samples except in the calamus of flight feathers, suggesting that the basic functions of these α-keratin genes are conserved in different feather types. Although feathers are composed mainly of feather-β-keratins, keratinocyte-β-keratins (basal β-keratins which diverged before the origin of feather-β-keratins) could also be important in determining whether feathers are plumulaceous or pennaceous as well as characteristics of the rachis, calamus, and barbs. Phylogenetic analysis and molecular dating show that the evolutionary origin of feathers might have occurred before the divergence of subfamily of feather-β-keratin genes, suggesting that the pennaceous feathers of some feathered dinosaurs could be composed of β-keratins rather than feather-β-keratins (Greenwold and Sawyer 2011).

These findings suggest that although the newly expanded gene family is critical for the evolution of a novel structure, existing genes have also been co-opted to play a significant role in these evolutionary innovations, so novel structures could have evolved before the appearance of new genes. Further analyses of the expression profiles between other newly expanded genes and not-expanded genes from a large panel of gene families may provide helpful information.

### In Situ Hybridization in Regenerating Follicles Using Specific Keratin Probes

**Fig. 5.—:**
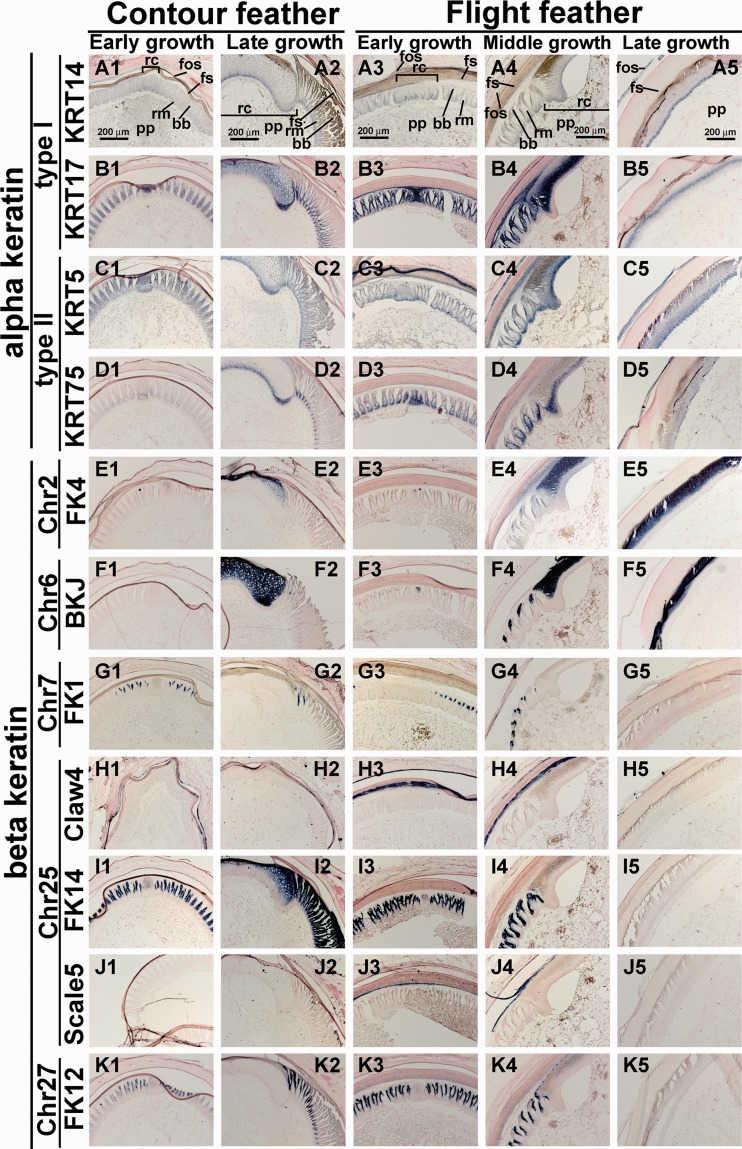
Section in situ hybridization of contour and flight feathers at different growth phases detected by specific α- and β-keratin probes. (*A1–B5*) Type I α-keratins. (*A1–A5*) KRT14. (*B1–B5*) KRT17. (*C1–D5*) Type II α-keratins. (*C1–C5*) KRT5. (*D1–D5*) KRT75. (*E1–K5*) β keratins. (*E1–E5*) FK4 on chromosome 2. (*F1–F5*) BKJ on Chr6. (*G1–G5*) Feather keratin 1 on Chr7. (*H1–H5*) Claw keratin 4 (Claw4) on Chr25. (*I1–I5*) Feather keratin 14 (FK14) on chromosome 25. (*J1–J5*) Scale keratin 5 (Scale5) on Chr25. (*K1–K5*) Feather keratin 12 (FK12) on Chr27. Columns 1 and 2, body contour feathers at early and late growth phase, respectively. Columns 3–5, wing flight feathers at early growth, middle growth, and late growth phase, respectively; bb, barbule; fs, feather sheath; fos, follicle sheath; pp, pulp; rc, rachis; rm, ramus.

To further examine the differential expression of α- and β-keratin genes in different feather follicles, we generated 50 specific α- and β-keratin antisense RNA probes, using the available sequences from 3’-UTR of mRNA, which are generally not conserved among paralogs as PCR targets. We present the in situ hybridization patterns for 11 of these probes (supplementary table S4, Supplementary Material online). These 11 probes include two Type I α-keratin genes, two Type II α-keratin genes, and seven β-keratin genes. The seven β-keratin genes include one feather-β-keratin 4 (FK4) on Chr2, one on Chr6 (BKJ), one feather β-keratin on Chr7 (FK1), three on Chr25 (one claw keratin, one feather keratin, and one scale keratin), and one feather-β-keratin on Chr27 (fig. 5).

The two Type I α-keratins displayed different expression patterns (fig. 5*A1–B5*). *KRT14* was expressed in the basal layer (fig. 5*A1–A5*), whereas *KRT17* in the suprabasal layer, in rachis and barbules (fig. 5*B1–B5*). The two Type II α-keratins also showed different patterns, with *KRT5* in the basal layer (fig. 5*C1–C5*), and *KRT75* in the rachis and ramus (fig. 5*D1–D5*); the patterns were the same as those in Ng et al. (2012), using a probe from the coding region. Both *KRT5* and *KRT75* were also expressed in the feather sheath and follicle sheath (fig. 5*C1–D5*).

β-keratins on Chr2, Chr6, and Chr7 showed different expression patterns (fig. 5*E1–G5*). FK4 on Chr2 was only expressed in the rachis. This keratin gene was strongly expressed in the rachis of late growth phase body contour feathers (fig. 5*E2*) and middle growth phase wing flight feathers (fig. 5*E4*). It was also expressed at a high level in the calamus during the late growth phase of wing flight feathers (fig. 5*E5*). BKJ on Chr6 was strongly expressed in the rachis of late contour feathers (fig. 5*F2*). However, it was expressed in both the rachis and ramus in the flight feathers (fig. 5*F4*). BKJ was also expressed at a high level in the calamus at the late growth phase of flight feathers (fig. 5*F5*). FK1 on Chr7 only was expressed in the feather branch, but not in the rachis, ramus, or calamus (fig. 5*G1–G5*). This expression pattern of Chr7_FK1 is consistent with BISK1 (barbule-specific keratin1), a β-keratin recently identified independently on chromosome 7 (Kowata et al. 2014).

The expression patterns of two feather keratins on Chr25 (fig. 5*I1–I5*) and Chr27 (fig. 5*K1–K5*) were similar. Both of them were expressed in the feather branches in early growth contour feathers (fig. 5*I1* and *K1*), EF (fig. 5*I3* and *K3*) and MF (fig. 5*I4* and *K4*). However, different expression patterns could be found in late growth contour feathers (fig. 5*I2* and *K2*). FK14 on Chr25 was expressed in the rachis, ramus, and all of the feather branches (fig. 5*I*2), whereas FK12 on Ch27 only was expressed in the ramus (fig. 5*K*2). Both claw- and scale-β-keratins on Chr25 were expressed in the feather sheath and follicle sheath (fig. 5*H1–H4* and *J1–J4*), but not inside the feather follicle. We did not detect their expression in the late grow flight feather (fig. 5*H5* and *J5*).

These results demonstrate that different α- and β-keratins have specific expression positions and levels. Different feather structures may use different keratin components to fine tune the structure for different functional purposes.

### Functional Characterization of Human *KRT5* Mutant Forms in Feather Development and Regeneration

To study the function of α-keratins in feather morphogenesis, we constructed three *KRT5* mutant forms that are related to human skin disease. In humans, *KRT5* mutations can cause the skin disease epidermolysis bullosa simplex. The three mutations were mimicked by deleting, respectively, Asn183 (mt1), Val170_Lys191 (mt2), and Arg464_Ala468 (mt3) of chicken K5, which correspond to Asn177, Val 164_ Lys 185, and Arg429_Ala433 of human K5, which may lead to epidermolysis bullosa herpetiformis, Dowling-Meara type (Rugg et al. 1999; Kemp et al. 2005; Kang et al. 2010) (supplementary table S7, Supplementary Material online).

**Fig. 6.—:**
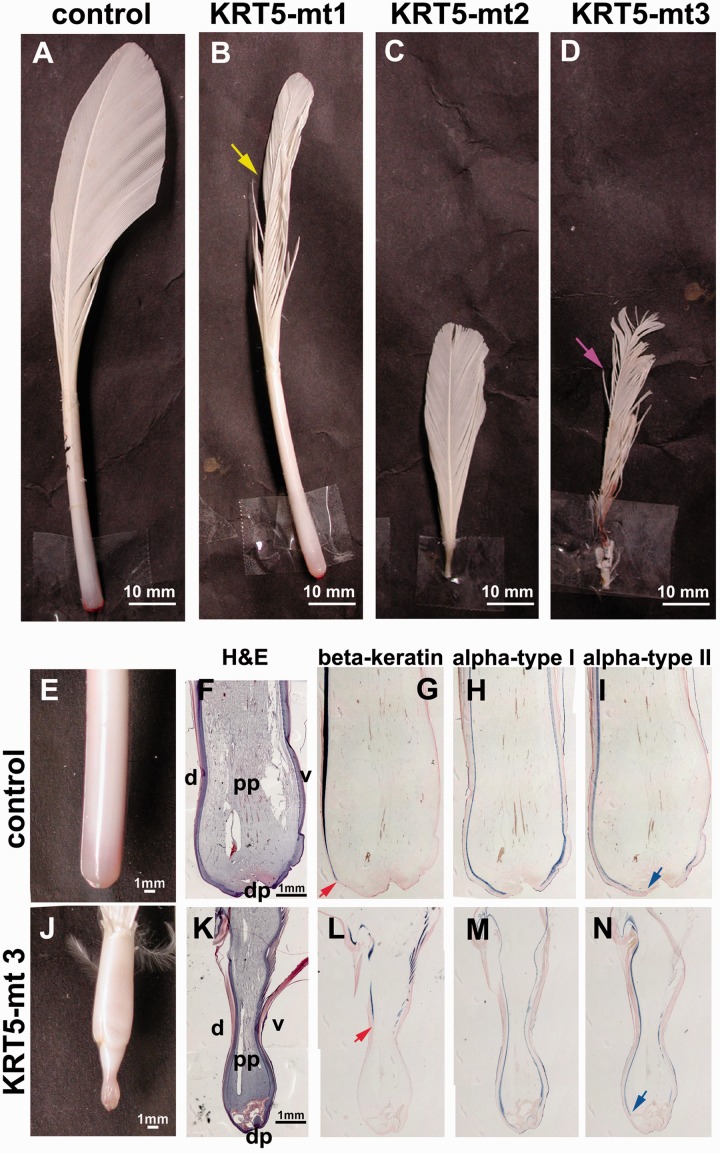
Functional study of *KRT5* mutant in adult flight feather regeneration. (*A–D*) Phenotype of feathers after misexpression of different KRT5 mutant forms in adult flight feathers after 40 days regeneration. (*A*) Control. (*B*) Mutant 1 (Asn183del). Yellow arrow indicates the branching defects. (*C*) Mutant 2 (Val170_Lys191del). (*D*) Mutant 3 (Arg464_Ala468del). Purple arrow indicates the severe branching defects. (*E–N*) Staining of control and mutant 3. Upper row, control; lower row, mutant form 3. (*E, J*) Bright view. (*F’, K’*) H&E staining. (*G–I*, *L–N*) In situ hybridization. (*G, L*) Common β keratin. (*H, M*) Common Type I α-keratin. (*I, N*) Common Type II α-keratin. Red and blue arrows indicate the distal point of common β keratin and common Type II α-keratin expression, respectively. d, dorsal; dp, dermal papilla. pp, pulp; v, ventral.

To examine whether the mutant forms can affect regeneration of adult feathers, we injected the RCAS virus into flight feather follicles, after plucking resting phase feathers. After 40 days of regeneration, control feathers were in the middle growth phase (fig. 6*A*). Regenerated feathers misexpressing mutant *KRT5* form 1 (KRT5-mt1) showed some branching defects (yellow arrow, fig. 5*B*), whereas regenerating feathers misexpressing either mutant forms 2 or 3 (KRT5-mt2, 3) stopped growing (fig. 6*C* and *D*). Mutant form 3 (KRT5-mt3) also showed severe branching defects (purple arrow, fig. 6*D*). We compared mutant form 3 (KRT5-mt3) feathers (fig. 5*E*) with normal regenerating feathers (fig. 6*J*). H&E staining of longitudinal sections showed shrinkage of the proximal feather end in the misexpressed sample (fig. 6*K*, compared with 6*F*). In situ hybridization of common keratin probes (fig. 6*G*–*I* and *L*–*N*) showed that β-keratin (fig. 6*L*, red arrow) and Type II α-keratin (fig. 6*N*, blue arrow) did not extend as proximal as the control (fig. 6*G*, red arrow and *I*, blue arrow). This result suggested that the *KRT5* mutant caused problems in proper assembly for both α- and β-keratins.

## Discussion

Our study contributes to the functional annotation of the majority of predicted chicken α- and β-keratins. We find that a large number of chicken α- and β-keratin genes are expressed in feather epithelial tissues. The expression patterns of these important corneous proteins have not been thoroughly studied in developing avian skin appendages. We combined RNA-seq and in situ hybridization data to correlate the spatially differential expression of these genes with different structures in different portions of the same type of feather as well as different types of feathers. We then functionally demonstrated that expression of mutant chicken α-keratin forms, corresponding to forms found in human disease, can have obvious phenotypes affecting the development and morphogenesis of chicken feathers. This shows that the chicken feather is an excellent model for studying the consequences and cellular mechanisms of these mutations.

### Corneous Feather Proteins

The tetrapod integument is formed by two classes of intermediate filament molecules termed Type I (acidic) and Type II (basic/neutral) α-keratins. These two types of α-keratin form an obligate heteropolymer that forms a cytoplasmic network. The epidermal α-keratins together with β-keratins function in the cornification process of the epidermal appendages of reptiles and birds (claws, scales, beaks, and feathers).

The β-keratin multigene family, on the other hand, is unrelated to the α-keratins and solely found in the genomes of sauropsids (reptiles and birds) and likely emerged after the divergence between the sauropsid and mammal lineages (Alibardi and Sawyer 2002; Sawyer et al. 2005; Wu et al. 2008; Greenwold and Sawyer 2010; Fraser and Parry 2011). β-keratins have been suggested to be renamed keratin-associated β-proteins (Toni et al. 2007; Alibardi and Toni 2008) because both reptilian and avian β-keratins are specialized proteins associated with the keratin meshwork that evolved in sauropsids and function as interkeratin matrix proteins or keratin-associated proteins (Toni et al. 2007; Alibardi et al. 2009; Dalla Valle et al. 2009).

Although the feather mainly consists of feather-β-keratins, which add much more rigidity than do α-keratins, cellular and biochemical studies have shown that α-keratins play an important role in the early formation of rachis, barbs, and barbules (Alibardi and Toni 2008). In amphibians, skin keratinization is less extensive because the skin is used as the primary respiratory organ (Alibardi 2003). Interestingly, the chicken genome contains even fewer α-keratin genes (33) than the anole genome (41) and the frog genome (36). In birds, β-keratin gene family expansion may have replaced some important roles of α-keratins during the formation of hard appendages, so that the remaining avian α-keratins might be irreplaceable in the formation of their skin appendages (Ng et al. 2012).

The molecular mechanisms for accumulating keratins in developing feathers are still largely unknown. Our study revealed that α- and β-keratins actually accumulate in different parts of the rachis and ramus. α-Keratins are generally expressed in the ventral part that is destined to become the medulla, whereas β-keratins are generally expressed in the dorsal part of the rachis and ramus that is destined to become the cortex. The identification of *KRT75*, encoding a Type II cytokeratin (basic), as a major determinant of normal feather structure suggests that α-keratins are critical for feather shaping (Ng et al. 2012).

Avian β-keratin gene expression has been characterized for the four major subfamilies (Greenwold and Sawyer 2010). For example, claw-β-keratins are expressed in developing claws and beaks (Whitbread et al. 1991; Wu, Jiang, et al. 2004), scale-β-keratins are found mainly in scutate scales (Walker and Bridgen 1976), and feather- and feather-like-β-keratins are found in both embryonic and adult feathers (Presland et al. 1989; Presland, Whitbread, et al. 1989). The tissue specificity of keratinocyte-β-keratins is unknown. Having so many feather-β-keratin genes on chromosomes 25 and 27 indicates intensive gene duplications that probably contributed to increased differences in textures and rigidity of feather types. Our analysis showed that feather-β-keratins from different chromosomes may be differentially expressed in various types of feathers and contribute to their specialized properties.

Previous studies already reported that the feather-like β-keratins are found not only in feathers but also in embryonic scales. Claw genes are expressed in both embryonic claws and feathers (Presland, Whitbread, et al. 1989; Whitbread et al. 1991). Our studies showed that although more than one subfamily is expressed in feather follicles, the expression patterns are specific in different feather regions. Numerous structural characteristics can be conveyed by a vast number of β-keratin dyad combinations, which are produced by high feather-β-keratin copy numbers and high N-terminus cysteine content. The numerous variants probably represent specialized feather β-keratins utilized in different types of feathers and contribute to the diversity of bird feathers. For instance, β-keratins on chromosomes 25 and 27 may contribute to the progressive maturation and hardening of barbule and barb cells, whereas β-keratins on chromosomes 2, 6, and 7 may be more suited for the calamus or the rachis in plumulaceous or pennaceous feathers.

### Keratinocyte β-Keratins

A total of 16 keratinocyte β-keratins have been identified in the chicken genome. They have low overall similarity to other avian β-keratins, and they diverge from one another within this newly found subfamily. The core-box that includes the 32 amino acids that make up the filament framework of β-keratins (Fraser and Parry 2008) is somewhat conserved, but the N- and C-termini vary significantly in both length and sequence. These keratinocyte-β-keratin genes were not well recognized and the expression profiles of most keratinocyte β-keratins in normal chickens were unexplored previously (Greenwold and Sawyer 2010, 2013).

We propose that the ancestral bird already had a diverse keratinocyte-β-keratin gene repertoire. The feather characteristics have evolved and diverged rapidly because of the divergent evolution of keratinocyte-β-keratins and the combinations with feather β-keratins. In birds, feather β-keratins are specifically expressed in feathers, but molecular evolution studies suggested that feather-β-keratins originated approximately 143 Ma, after the first appearance of pennaceous feathers in *Anchiornis* (∼155 Ma) (Greenwold and Sawyer 2011). We observed that most β-keratin genes are expressed in the feather follicle, although feather-β-keratins play more specialized roles in bird feather development. Therefore, the pleisiomorphic feathers of ancestral archosaurs also likely employed the full repertoire of β-keratins (Greenwold and Sawyer 2013).

### Chicken As a Model to Study Mechanism of Human Keratin Diseases

There are at least 54 functional α-keratin genes in the human genome (Hesse et al. 2004; Moll et al. 2008). Various combinations of α-keratin proteins are found in different tissues in which a Type I α-keratin couples with a Type II α-keratin to form a heterodimer which interacts with another heterodimer to form flexible, firm keratin intermediate filaments. The keratin filaments then assemble into a dense network which is critical for the mechanical integrity and stability of epithelial cells and tissues (Fuchs and Cleveland 1998). In addition, some α-keratins also have regulatory functions and are participated in intracellular signaling pathways, for example, cell growth and proliferation, cell motility, wound healing, protection from stress, and apoptosis (Pan et al. 2013).

Mutations in α-keratin genes alter the structure of α-keratin proteins, which may interfere them from constructing an effective structural framework of cells (Fuchs and Cleveland 1998). Cells are easily damaged without this dense network, making tissues less resistant to friction and minor trauma (Omary et al. 2004). Mutations in at least 22 α-keratin genes are known to cause human diseases (keratinopathies) affecting the skin, nails, hair, cornea, liver, and related tissues (Chamcheu et al. 2011), resulting in cardiomyopathies, skin fragility conditions, and premature aging (Pan et al. 2013).

Most of the available keratin mouse models were generated by taking advantage of conventional gene-targeted disruption or deletion (Magin 1998; Vijayaraj et al. 2007; Chen and Roop 2008). However, this strategy necessitates germ line introduction of mutated gene copies to reveal the molecular mechanisms by which mutations lead to cell and tissue fragility. Our study showed that chicken feathers can be an outstanding model to study keratin function because RCAS transgenic experiments are much easier to perform and less time-consuming and costly than generating knock-in mice. We recently showed that an α-keratin mutation, a 23 amino-acid deletion in a conserved region of *KRT75*, caused the Frizzle chicken phenotype (Ng et al. 2012). In this study, we successfully misexpressed *KRT5* carrying the mutations found in humans and revealed their divergent phenotypes. Furthermore, our strategy also offers the possibility to avoid embryonic or neonatal lethality in some of the keratin-deficient mice.

## Conclusions

Recent advances in deep sequencing technologies allowed us to conduct the first comprehensive RNA-seq analysis of keratin genes expressed in feather epithelium. We performed transcriptomic analyses of different feather follicles at different developmental stages. These analyses and in situ hybridization revealed different keratin expression patterns in regenerating feather follicles. Finally, functional analysis using mutant forms based on human *KRT5* mutations demonstrated the potential to use the chicken as a model to study keratin-based diseases. Further studies are needed to analyze gene regulation for the rapid production of β-keratins, the polymerization of different β-keratins, and their association with the cytoskeleton present in feather cells.

## Supplementary Material

Supplementary tables S1–S7 and figures S1–S7 are available at *Genome Biology and Evolution* online (http://www.gbe.oxfordjournals.org/).

Supplementary Data
