# Supplementary material for: Genomic Organization, Transcriptomic Analysis, and Functional Characterization of Avian α- and β-Keratins in Diverse Feather Forms
Source: Genome Biol Evol. 2014 Aug 24;6(9):2258–73. doi: 10.1093/gbe/evu181 (PMC4202321; doi:10.1093/gbe/evu181)
Supplement: Supplementary Data [file supp_6_9_2258__index.html]

Genomic organization, transcriptomic analysis, and functional characterization of avian α- and β-keratins in diverse feather forms — Genomic Organization, Transcriptomic Analysis, and Functional Characterization of Avian α- and β-Keratins in Diverse Feather Forms — Supplementary Data 

# Genomic Organization, Transcriptomic Analysis, and Functional Characterization of Avian α- and β-Keratins in Diverse Feather Forms

## Supplementary Data

files

**Files in this Data Supplement:**

- Supplementary Data - pdf file
- Supplementary Data - xlsx file
- Supplementary Data - xlsx file
