# Supplementary material for: Genomic Organization, Transcriptomic Analysis, and Functional Characterization of Avian α- and β-Keratins in Diverse Feather Forms
Source: Genome Biol Evol. 2014 Aug 24;6(9):2258–73. doi: 10.1093/gbe/evu181 (PMC4202321; doi:10.1093/gbe/evu181)
Supplement: Supplementary Data [file supp_evu181_Supplementary_figure_S1-S7_Table_S4-S5.pdf]

Supplementary figure S1

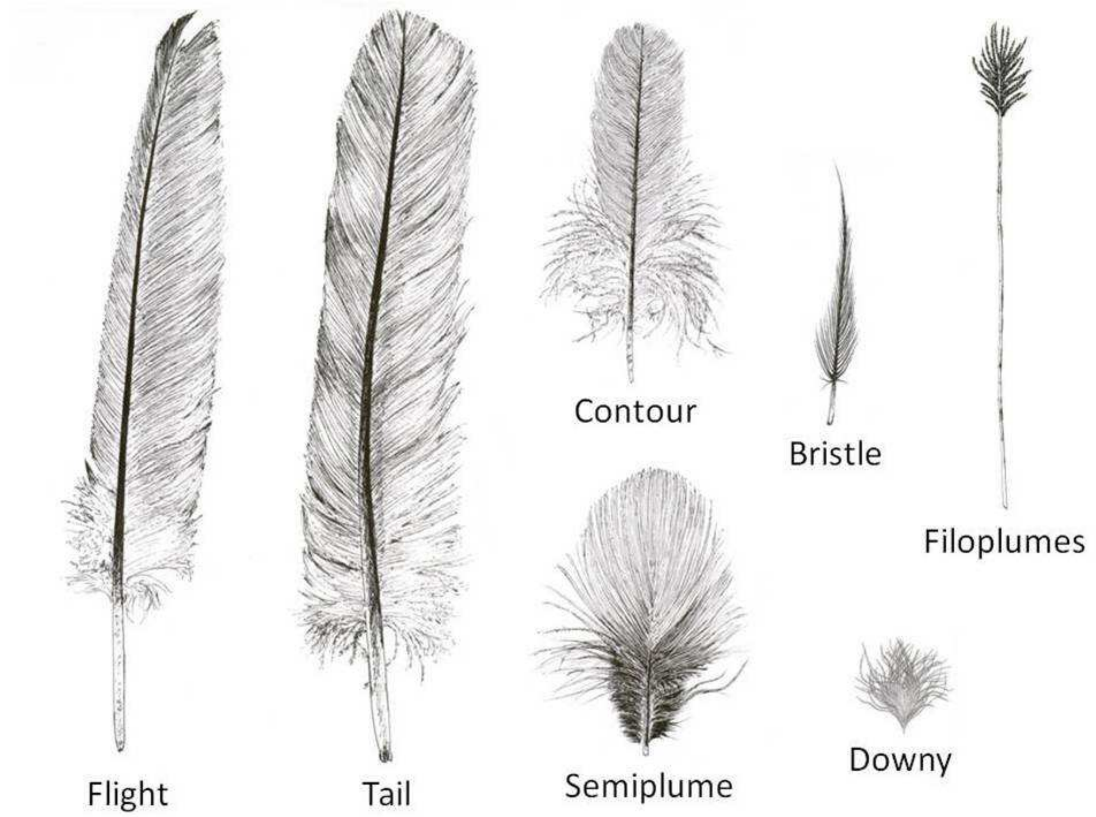

Supplementary figure S2

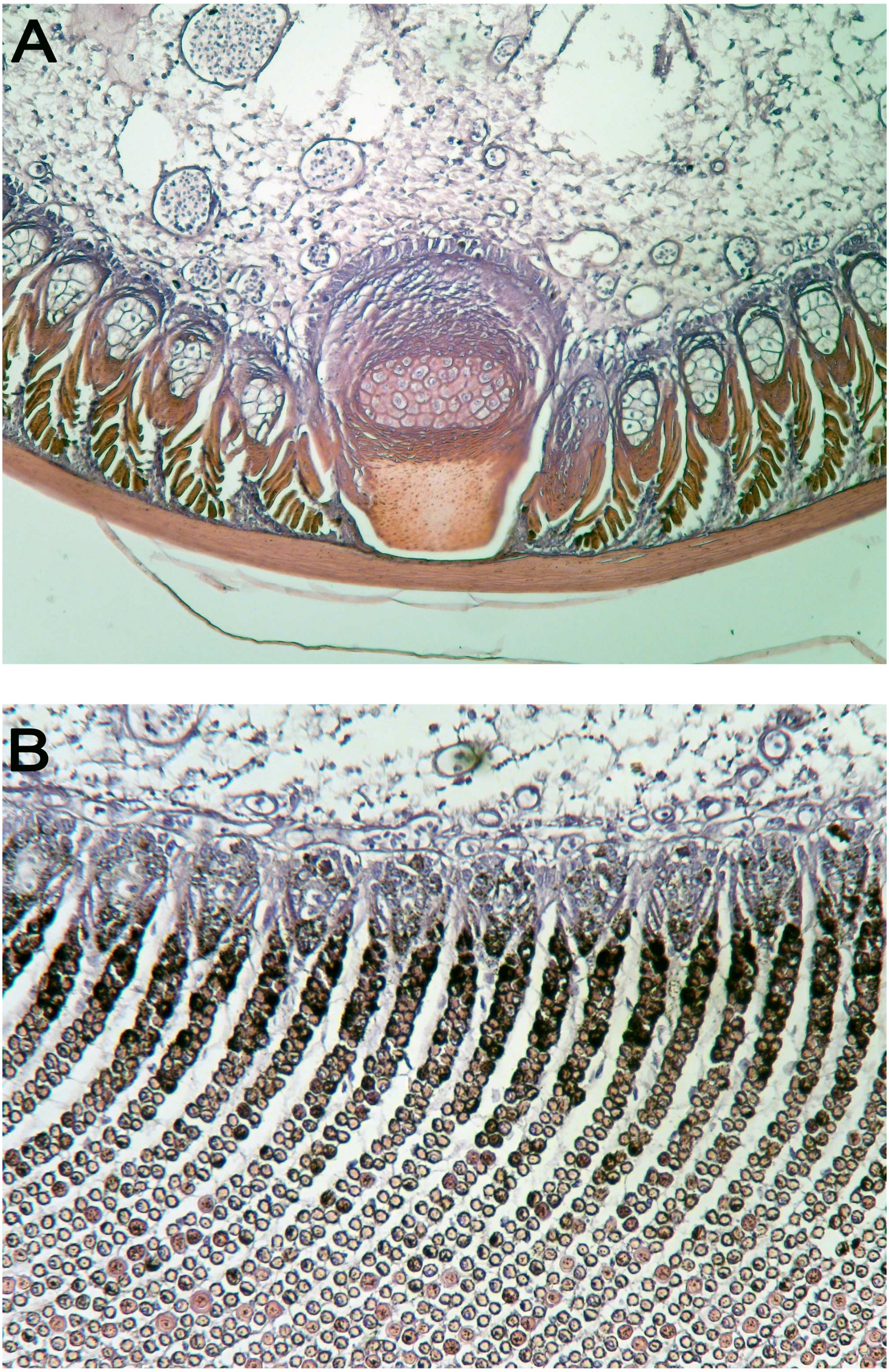

Supplementary figure S3

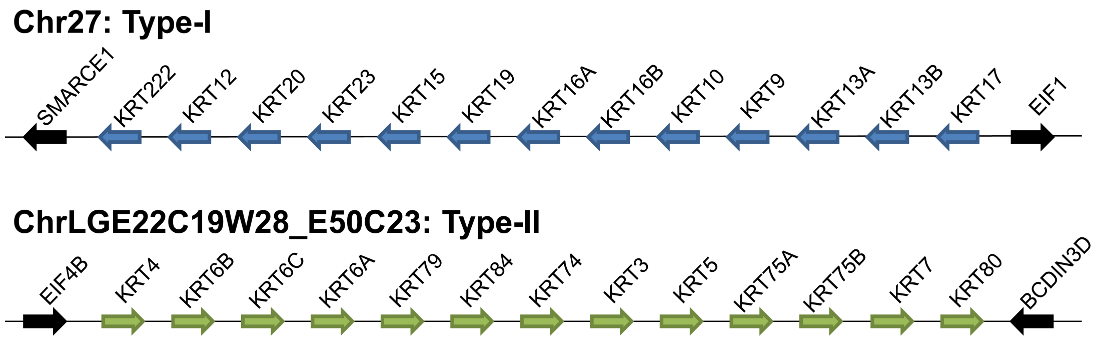

Supplementary Table S4

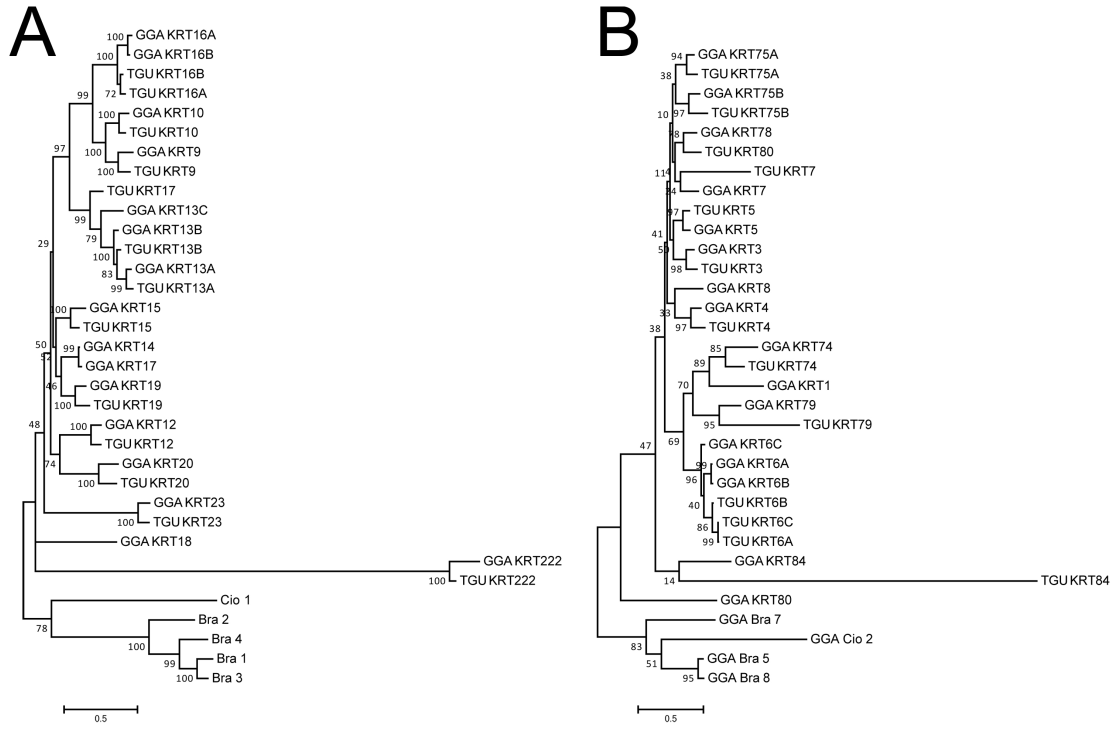

Supplementary figure S5

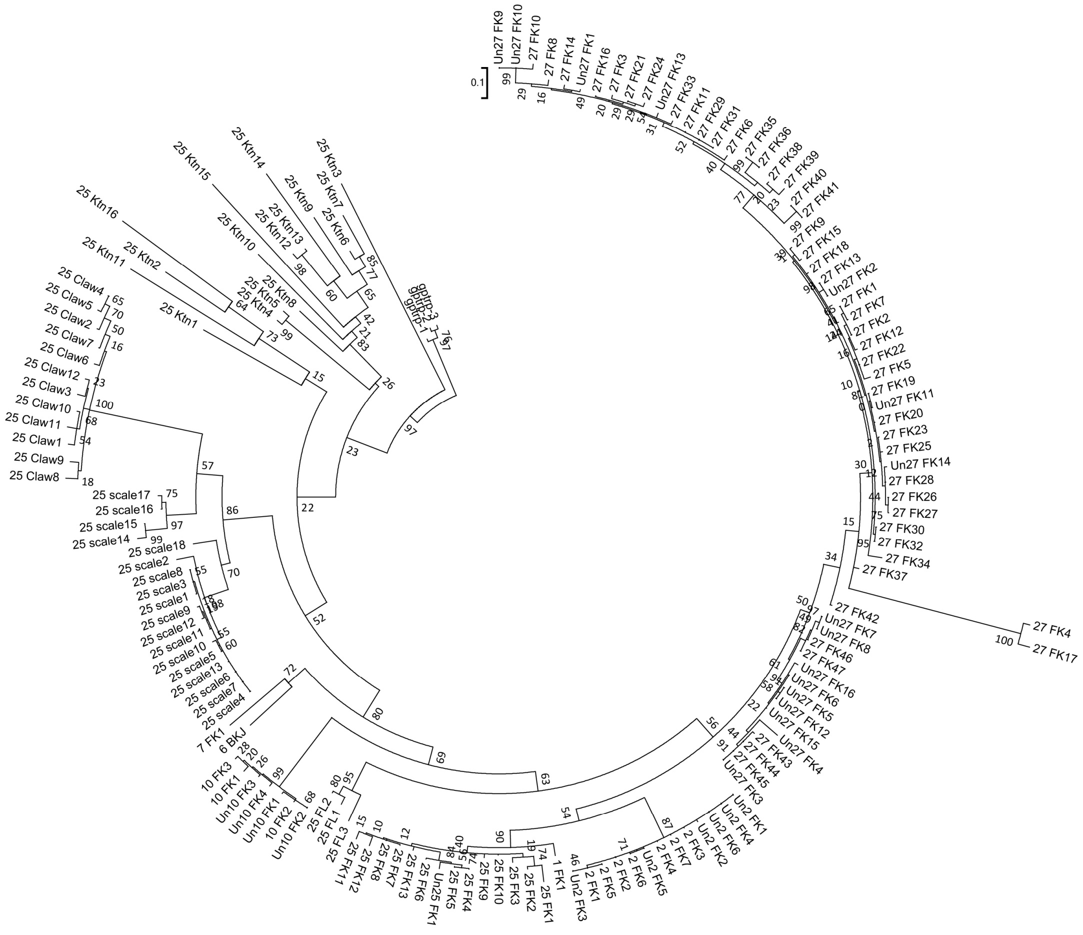

Supplementary figure S6

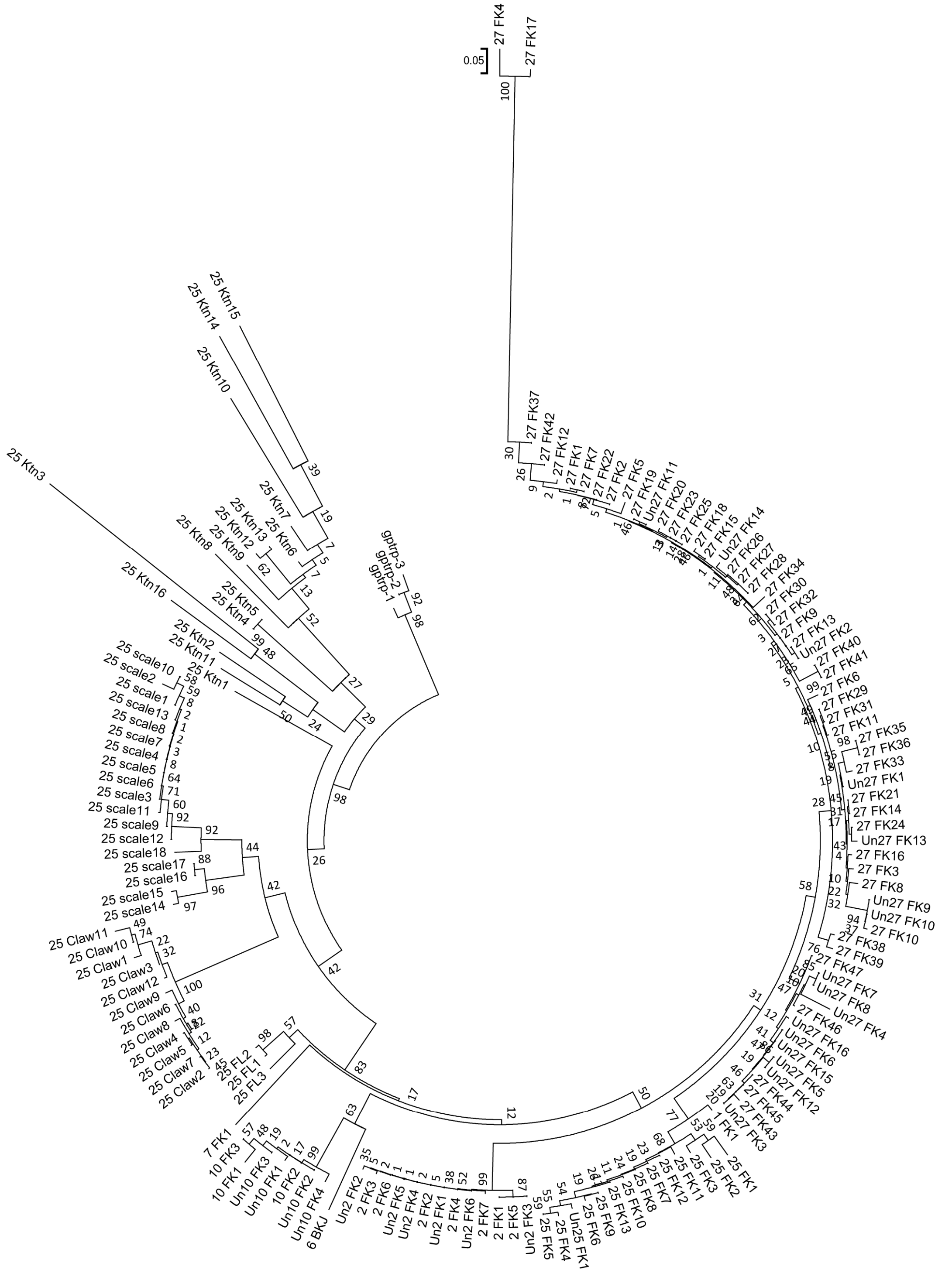

Supplementary figure S7

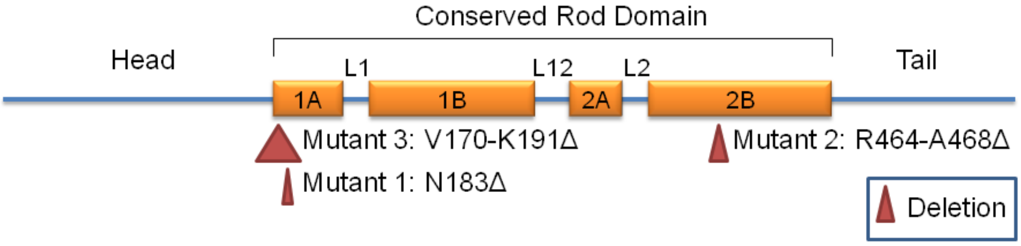

Supplementary Table S4. List of PCR primers for generating specific and common keratin probes

	Forward primer	Backward primer	PCR size (bp)
Type I alpha keratin			
KRT14	CTCATCCCGTGAGCAGATG	GCTTTATTAAATGTGTACAGAATGCAC	173
KRT17	CGGGCTAGGAGATGACACAG	CATCAGGCAGAAGCACAGTT	251
Type II alpha keratin			
KRT5	GCAACGTGCTGTCTTACCAA	GATGTGAGTAGGGGCTTCCA	393
KRT75	CTCCCTCACCAGAAAACACC	GACAAACACCAGAGAGTGAAGAGA	302
Beta keratin			
FK4 in chromosome 2	GCTGTCTGGAAACATGAGCA	ACCAATGCACTTGTTGGTGA	280
BKT3 in chromosome 6	CACCTGCCTGTGCATGAG	CCAAAGCTGATGCTCAATGT	255
FK1 in chromosome 7	AGGAAAAGCAAAGCACAGGA	GGACAAAACGACCCCTACAA	312
Claw4 in chromosome 25	CTCTGTCCGTGGTTGAAGAAG	AGAGGGCAGAGGGACAGG	230
FK14	TGAGGTGGACATCCTGTGAA	ACAATGGGATGCCTGACTTC	329

in chromosome 25			
Scale5 in chromosome 25	ATCTCACATGAAGGCCCAAG	TGTTTCCAGACAGTTCCAGAGA	309
FK12 in chromosome 27	GCCATGATCCTGGTGAAATC	AGCTCATGCAAGGCTTGTG	316
Common Type I alpha keratin	ACAATGCAGAACCTCAACGA	CAATCTGCATCTCCAGGTCA	367
Common Type II alpha keratin	CGACAACAAATTTGCCTCCT	CATCTGCCTTGGCCTGTAGT	359
Common beta keratin	ATGTCCTGCTCCAACCTC	GGGGAAGGAGCTGAGGAT	156

Supplementary Table 5

**Human homolog**	**protein variant**	**Disease**	**Chicken homolog**	**protein variant**
KRT5	p.V164-K185Δ	Epidermolysis bullosa simplex, Dowling-Meara type	KRT5	p.V170-K191Δ
	p.N177Δ	Epidermolysis bullosa simplex, Dowling-Meara type		p.N183Δ
	p.R429-A433Δ	Epidermolysis bullosa simplex, Dowling-Meara type		pR464-A468Δ
